# Opposing Roles of Calcium and Intracellular ATP on Gating of the Purinergic P2X2 Receptor Channel

**DOI:** 10.3390/ijms19041161

**Published:** 2018-04-11

**Authors:** Milos B. Rokic, Patricio Castro, Elias Leiva-Salcedo, Melanija Tomic, Stanko S. Stojilkovic, Claudio Coddou

**Affiliations:** 1 Section on Cellular Signaling, National Institutes of Child Health and Human Development, NIH, Bethesda, MD 20892, USA; milos.rokic@nih.gov (M.B.R.); eliasleiva@gmail.com (E.L.-S.); stojilks@mail.nih.gov (S.S.S.); 2 Departamento de Ciencias Biomédicas, Facultad de Medicina, Universidad Católica del Norte, Coquimbo 1781421, Chile; pacastrom@gmail.com; 3 Laboratory of Developmental Physiology, Department of Physiology, Faculty of Biological Sciences, Universidad de Concepción, Concepción 4030000, Chile; 4 Departamento de Biología, Facultad de Química y Biología, Universidad de Santiago de Chile, Santiago 9170022, Chile; 5 Centro para el Desarrollo de Nanociencias y Nanotecnología (CEDENNA), Santiago 9170022, Chile

**Keywords:** purinergic receptor channels, desensitization, ATP, calcium, allosteric, covalent

## Abstract

P2X2 receptors (P2X2R) exhibit a slow desensitization during the initial ATP application and a progressive, calcium-dependent increase in rates of desensitization during repetitive stimulation. This pattern is observed in whole-cell recordings from cells expressing recombinant and native P2X2R. However, desensitization is not observed in perforated-patched cells and in two-electrode voltage clamped oocytes. Addition of ATP, but not ATPγS or GTP, in the pipette solution also abolishes progressive desensitization, whereas intracellular injection of apyrase facilitates receptor desensitization. Experiments with injection of alkaline phosphatase or addition of staurosporine and ATP in the intracellular solution suggest a role for a phosphorylation-dephosphorylation in receptor desensitization. Mutation of residues that are potential phosphorylation sites identified a critical role of the S363 residue in the intracellular ATP action. These findings indicate that intracellular calcium and ATP have opposing effects on P2X2R gating: calcium allosterically facilitates receptor desensitization and ATP covalently prevents the action of calcium. Single cell measurements further revealed that intracellular calcium stays elevated after washout in P2X2R-expressing cells and the blockade of mitochondrial sodium/calcium exchanger lowers calcium concentrations during washout periods to basal levels, suggesting a role of mitochondria in this process. Therefore, the metabolic state of the cell can influence P2X2R gating.

## 1. Introduction

Extracellular ATP-gated P2X receptor channels (P2XRs) are composed of three homologous or heterologous subunits; in mammals seven subunits, designated P2X1-7, have been identified. The gating of these channels is very complex and usually consists of three phases: a rapid rise in inward current caused by binding of ATP, termed activation; a progressively developing decay of the current in the continued presence of ATP, termed desensitization; and a relatively rapid decay in the current after washout of the ligand, termed deactivation [1]. Desensitization kinetics is receptor-specific, from the fast desensitizing P2X1R and P2X3R to the slow desensitizing P2X2R and P2X4R, and even the non-desensitizing P2X7R, which exhibits increased currents upon sustained activation [2].

Numerous studies were focused on structural elements accounting for receptor-specific desensitization pattern. Briefly, the cysteine-rich head domain located above the ATP-binding cleft of P2X1R [3] and the negatively charged aspartate residues located below the ATP-binding site of P2X3R [4] play important roles in the transition from the open to the desensitized state. The transmembrane tyrosine residues stabilize the desensitized state [5]. Interactions between the ectodomain and regions around the transmembrane domains have also been implicated as being relevant in receptor desensitization [6]. It is also well established that the N- and C-terminal domains provide the structural elements involved in controlling the kinetics of receptor desensitization. These studies included work with splice forms of P2X2Rs [7,8,9], and mutagenesis studies focused on identification of the residues that account for those effects [10,11]. Experiments with chimeric P2X2R and P2X1R also suggested a role of intracellular N-terminus in receptor desensitization [12]. In particular, the most attention has been devoted to the conserved threonine and tyrosine residues located in the N-terminus [13,14,15,16,17,18].

P2XRs are highly regulated channels and can respond to changes in their extracellular and intracellular environment, which further contributes to the complexity in their gating properties [2]. This includes the roles of bath and intracellular calcium in receptor gating; its main effect is observed in a millimolar concentration range causing inhibition of agonist-induced currents [19], but it has also been suggested that Ca^2+^ facilitates recovery from desensitization of P2X3Rs [20]. Recently, we identified a novel aspect of P2X2R desensitization, termed use-dependent desensitization (UDD), which is manifested by a progressive increase in rates of P2X2R desensitization during repetitive application of ATP [21]. This process was observed in whole-cell recordings from HEK293 cells expressing both splice forms of the rat, mouse, and human receptors, termed P2X2aR and P2X2bR [22]. We also found that this process depends on calcium influx and that domain calcium is sufficient to establish UDD in the whole-cell recording [22]. However, the fact that nanomolar calcium concentrations are sufficient to develop UDD strongly suggest that other molecules present in the intracellular environment are also important to counteract calcium effects and therefore to regulate P2X2R gating.

Here, we utilized electrophysiology and mutagenesis to test the relevance of intracellular ATP and calcium in P2XRs gating, an issue that has been previously addressed for other plasma membrane channels [23,24,25,26], but not for P2XRs, that are gated by extracellular ATP. We found that P2X2R gating is dependent on both intracellular ATP and calcium, having these ligands opposite effects in P2X2R desensitization, with calcium accelerating and ATP impeding desensitization. The action of intracellular calcium is allosteric, whereas intracellular ATP acts covalently, as the phosphate source for receptor phosphorylation. We also identified the relevance of the C-terminal S363 residue for intracellular ATP regulation. These data suggest that P2X2Rs represent an important functional crosslink between extracellular and intracellular ATP and calcium signaling and provide integration of cellular responses to these ligands.

## 2. Results

### 2.1. The P2X2R Current Response Pattern Depends on Recording Conditions

**Figure 1:**
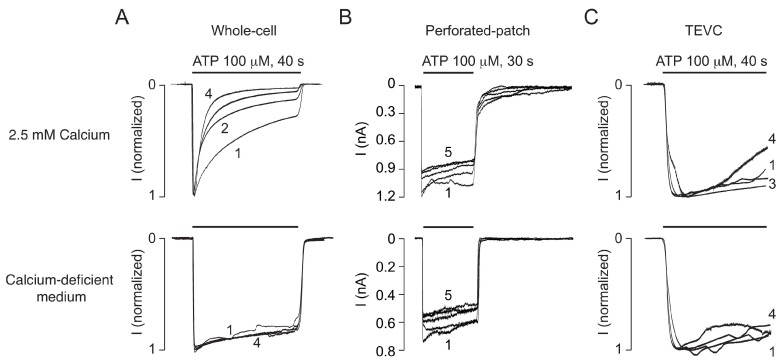
Dependence on recording conditions of the pattern of rat P2X2a receptor (P2X2aR) desensitization to repetitive ATP application. (**A**) Whole-cell recording in HEK293 cells. Traces of currents induced by repetitive application of 100 μM ATP for 40 s with 4 min washout periods for cells bathed in calcium-containing medium (2.5 mM; top traces) or in calcium-deficient medium (0.09 mM calcium; bottom traces) at a holding potential of −60 mV are shown. The progressive increase in the rates of receptor desensitization is termed use-dependent desensitization (UDD); (**B**) Perforated patch-clamp recording in HEK293 cells. Notice that there is no difference in the rate of receptor desensitization during repetitive agonist application (100 μM ATP for 30 s with 4 min washing periods) in cells bathed in calcium-containing (top) or calcium-deficient (bottom) medium; (**C**) Two-electrode voltage clamp (TEVC) recording in *Xenopus* oocytes. The currents induced by four consecutive ATP applications in oocytes bathed in calcium-containing (top) or calcium-deficient (bottom) medium are shown. In (**A**,**C**), the data shown are normalized representative recordings. In all panels, traces shown are representative of at least five similar experiments. In this and the following figures, horizontal black bars indicate duration of ATP application, and the numbers indicate the order of each ATP application.

We recently reported that P2X2R undergoes a progressive calcium-dependent desensitization during repetitive ATP application, a phenomenon termed UDD. This process was observed in whole-cell recordings from HEK293 cells expressing both splice forms P2X2aR and P2X2bR of the rat, mouse, and human receptors [22]. Figure 1A shows an example of UDD using rat P2X2aR. The cells were stimulated four times for 40 s with 100 μM ATP followed by 4 min washout periods in calcium-containing (2.5 mM) or calcium-deficient (0.09 mM) medium. For the cells bathed in calcium-containing medium (top), but not in calcium-deficient medium (bottom), there was pronounced receptor desensitization during the first ATP application and a further increase in the rate of receptor desensitization with each subsequent ATP application. In those experiments, the peak amplitude of currents using calcium-containing or calcium-deficient medium were not significantly different (5.4 ± 0.3 μA vs. 5.0 ± 0.7 nA; *n* = 8–11). UDD was independent of the method of transfection; it was observed in both the polymer-based and lipid-based transfection experiments.

However, when we used perforated patch-clamp recording in HEK293 cells, no difference in the rate of receptor desensitization was observed during five consecutive agonist applications (Figure 1B) in cells bathed in calcium-containing (top) or calcium-deficient (bottom) medium. The lack of the ability of repetitive ATP application to generate UDD in calcium-containing medium was also observed in two-electrode voltage clamp TEVC recording in P2X2aR-expressing *Xenopus* oocytes. Figure 1C shows that the profiles of the currents induced by four consecutive ATP applications in oocytes bathed in calcium-containing (top) and calcium-deficient (bottom) medium were comparable. These results indicated that calcium influx affects the rate of P2X2aR desensitization in the whole-cell patch clamp configuration, but not in the recording configurations that preserve the intracellular cytosol.

**Figure 2:**
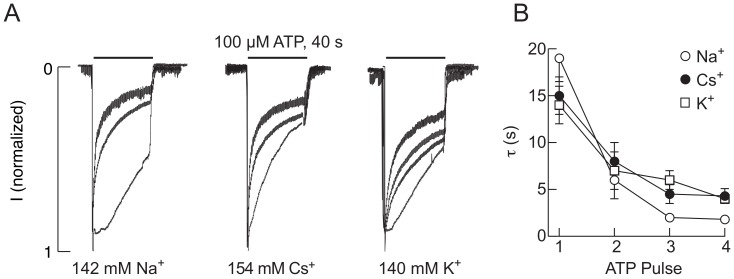
UDD is present and comparable in various intracellular ionic environments. (**A**) The traces shown are representative of whole-cell recordings in HEK293 cells with the intrapipette medium containing sodium (left), cesium (middle), and potassium (right) chloride. In all cases, 2.5 mM calcium was present in the extracellular medium, and the holding potential was −60 mV; (**B**) Mean ± SEM values of the rate of receptor desensitization (τ) derived from a monoexponential fit for sodium, cesium, and potassium-based intracellular solutions (*n* = 10–14 per group).

The experiments shown in Figure 1A, top panels, were performed using an intrapipette solution containing 142 mM NaCl, 10 mM EGTA, and 10 mM HEPES and extracellular medium containing 142 mM NaCl, 3 mM KCl, 1 mM MgCl, 2.5 mM CaCl_2_, 10 mM glucose, and 10 mM HEPES. To examine whether the equimolar concentrations of Na^+^ in extracellular and intracellular media specifically account for development of UDD, in additional experiments, KCl (140 mM) or CsCl (154 mM) was substituted for the intracellular NaCl. Figure 2A shows that UDD also developed in the presence of the K^+^- and Cs^+^-containing intracellular solutions. Furthermore, Figure 2B shows that the rates of receptor desensitization were quantitatively comparable in all three experimental conditions. Similar to the results of the experiments using the Na^+^-containing intrapipette solution (Figure 1A, bottom panel). Thus, Ca^2+^ influx is critical for development of UDD independent of the nature of the major intrapipette cation and independent of the expression system and/or gene transfection technique.

### 2.2. Endogenous P2X2R-Mediated Currents also Exhibit UDD

**Figure 3:**
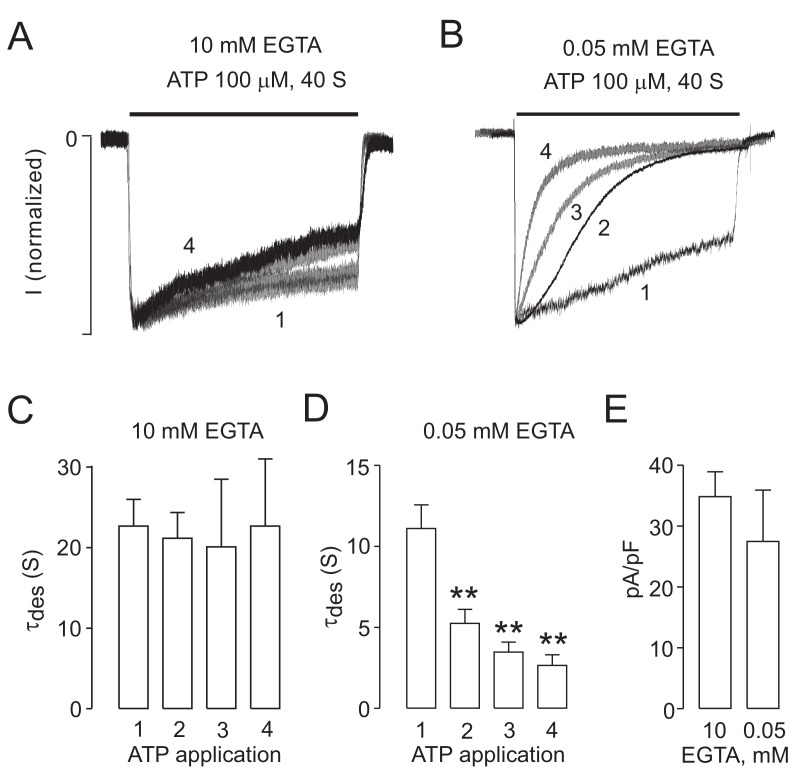
Use-dependent desensitization on PC12 cells. (**A**) Representative tracings of a single PC12 cell challenged with four successive applications of 100 μM ATP and the intracellular solution containing 10 mM EGTA; (**B**) The same experiment in other PC12 cells in which the intracellular solution includes 0.05 mM EGTA; UDD is developed under such conditions; (**C**,**D**) Summary of the desensitization constants in four consecutive ATP applications in cells clamped with an intracellular solution containing 10 mM (**C**) or 0.05 mM (**D**) EGTA; (**E**) Current densities for both conditions. ** *p* < 0.01, test. Mann-Whitney test. *n* = 4–8.

The experiments of UDD shown so far are all performed in heterologous systems that overexpress the recombinant rat P2X2R. We next tested if this phenomenon is also observed in cells that endogenously express the P2X2R. For that purpose we used undifferentiated PC12 cells, the source of the first rat P2X2R cDNA cloned [27]. In whole-cell recordings, these cells exhibited robust, slow-desensitizing ATP currents, resembling the P2X2R desensitization profile (Figure 3A), with an average amplitude of 302 ± 43 pA, that is more than 10-fold smaller that the amplitudes obtained in P2X2R-expressing HEK293 cells. The use of an intracellular solution with 10 mM EGTA resulted in slow desensitizing currents with no further increases in receptor desensitization (Figure 3A), but when EGTA was lowered to 0.05 mM EGTA the endogenous currents immediately started to develop UDD (Figure 3B). This observation was confirmed when we analyzed the changes in desensitization rates in both conditions (Figure 3C,D). The variations on intracellular EGTA did not affect the current densities obtained in each condition (Figure 3E).

### 2.3. Intracellularly Applied ATP Abolishes UDD

**Figure 4:**
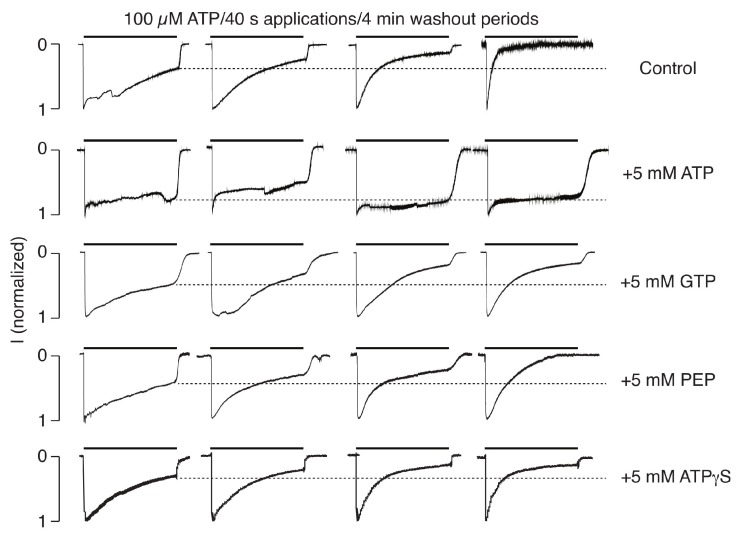
Intracellular ATP, but not GTP, phosphoenolpyruvate (PEP), or ATPγS, blocks receptor desensitization in the whole-cell recordings performed in HEK293 cells expressing P2X2aR. The cells were dialyzed with ATP, GTP, PEP, or ATPγS for 7 min before the start of the extracellular ATP application, and the holding potential was −60 mV. The horizontal dotted lines illustrate the peak in the current response at the end of the first ATP application. Notice that UDD is preserved in all cases except for the cells filled with 5 mM ATP.

Performing whole-cell recording is associated with dilution of numerous intracellular factors, including ATP. In order to test the potential role of intracellular ATP on P2X2R gating, the whole-cell recording was done in HEK293 cells with the potassium-containing intrapipette solution without and with 5 mM ATP. In contrast to the control conditions, which showed typical UDD, there was a loss of the receptor UDD in the cells filled with 5 mM ATP-containing medium (Figure 4). In the presence of ATP, there was no difference in the pattern of currents in the cells bathed in calcium-containing and calcium–deficient medium, further confirming that calcium-dependent UDD does not occur in cells filled with 5 mM ATP.

We also examined whether GTP, phosphoenolpyruvate, or ATPγS could substitute for ATP in blocking the calcium-dependent desensitization of P2X2R in the whole-cell recording. GTP is a source of energy and an activator of substrates in metabolic reactions, similar to ATP, but is more specific due to the difference in the purine ring structure. However, GTP could not substitute for ATP in blocking UDD (Figure 4). The phosphoenolpyruvate anion has the phosphate bond with the highest free energy found in living organisms and is involved in glycolysis and gluconeogenesis but was unable to block development of UDD (Figure 4). ATPγS is a non-hydrolyzable ATP analog that has agonist activity, i.e., can substitute for ATP in the activation of P2XRs, but this analog could not mimic the regulatory role of ATP in the channel gating (Figure 4). Together, these results indicate that ATP does not affect P2X2R gating directly by binding to an intracellular binding site but, presumably, by the ATP-dependent and kinase-mediated phosphorylation of the channels.

**Figure 5:**
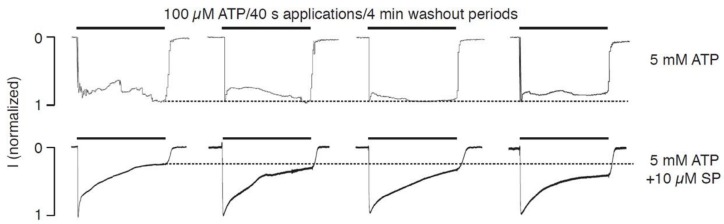
Potential role of protein kinases in the intracellular ATP effects on receptor desensitization; whole-cell recordings in HEK293 cells expressing P2X2aR at a holding potential of −60 mV. (**Top panels**) The loss of receptor desensitization in cells filled with 5 mM ATP-containing medium; (**Bottom panels**) Restoration of receptor desensitization in cells filled with a medium containing 5 mM ATP plus 10 μM staurosporine (SP). Notice the lack of UDD in the presence of SP. In both experiments, the cells were dialyzed for 7 min before the extracellular ATP was applied. The horizontal dotted lines indicate the peak amplitude of the current at the end of the first pulse of extracellular ATP.

**Figure 6:**
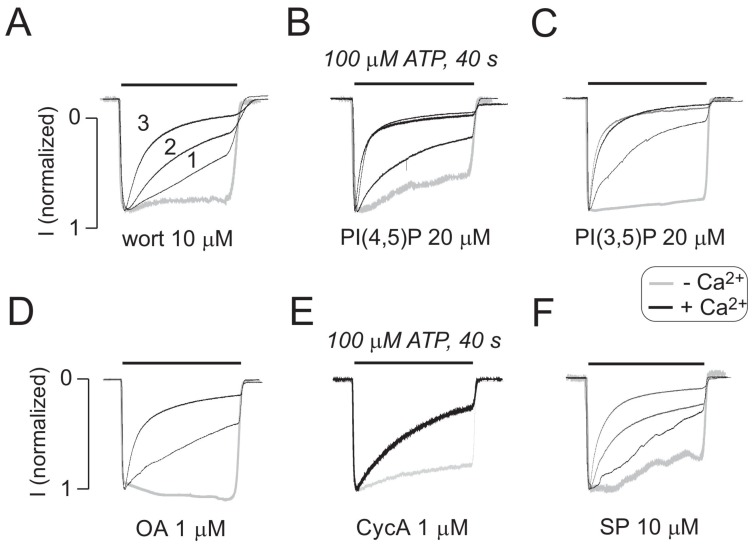
Independence of UDD of the status of kinases, phosphatases, and phosphoinositides in the absence of intracellular ATP. The results show whole-cell recordings from P2X2aR-expressing HEK293 cells clamped at −60 mV and containing kinase and phosphatase inhibitors or activators in the absence (gray traces) or in the presence (black traces) of 2 mM extracellular calcium. (**A**–**F**) The lack of effects of wortmannin (WT), an inhibitor of PI_3_ and PI_4_ kinases (**A**), the phosphoinositides PI(4,5)P_2_ (**B**) and PI(3,5)P_2_ (**C**), the phosphatase PP2B inhibitor okadaic acid (OA) (**D**), the calcineurin inhibitor cyclosporin A (CycA) (**E**) and staurosporine (SP), a nonselective kinase inhibitor at the concentration used (**F**), on the development of UDD during repetitive agonist application. The cells were dialyzed for 7 min before the start of the experiment, and the washout periods between ATP applications were 4 min each. The recordings shown are representative of at least 3 different experiments.

To address this hypothesis, we examined the effects of intracellular ATP on the pattern of current signaling in the absence and presence of 10 μM staurosporine, a prototypical ATP-competitive kinase inhibitor that binds to many kinases with high affinity but very little selectivity [28]. In contrast to the results for ATP alone, in the presence of ATP and staurosporine, there was pronounced receptor desensitization, but UDD was lost (Figure 5), which supported the hypothesis. Intracellular calcium also affects the status of phosphoinositides through various pathways [29], and these compounds also contribute to the control of P2XR gating [30,31,32,33,34]. We assessed the potential role of phosphoinositides in P2X2R desensitization in the absence of intracellular ATP by adding the phosphoinositides PI(4,5)P, PI(3,5)P (Figure 6A,B) to the intracellular solution. Additionally, we inhibited PI3K and PIK4K with wortmannin (wort, Figure 6C). The potential role of protein phosphatases in the absence of intracellular ATP was also tested by adding the PP2B and calcineurin inhibitors, okadaic acid and cyclosporin A, respectively (Figure 6D,E). In all conditions tested, P2X2R-mediated currents preserved the Ca^2+^-induced increase in receptor desensitization. As expected, the effects of staurosporine shown in Figure 5 were not observed in experiments lacking intracellular ATP (Figure 6F).

**Figure 7:**
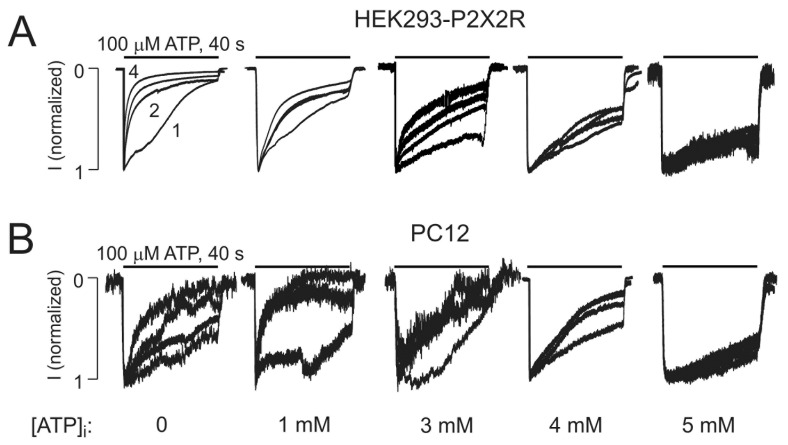
Intracellular ATP concentration-response experiments. (**A**,**B**) Traces of currents induced by repetitive application of 100 μM ATP for 40 s with 4 min washout periods for cells bathed in calcium-containing medium with the intracellular solution containing 0, 1, 3, 4 or 5 mM ATP in HEK293 cells transfected with the P2X2R cDNA (**A**) or in PC12 cells (**B**) that endogenously express the P2X2R. Recordings were performed in at least three different cells for each condition.

Next, we tested different intracellular ATP concentrations to elucidate the range at which ATP can prevent UDD. The experiments were performed in both HEK293 cells overexpressing the P2X2R and in PC12 that endogenously express this receptor. In both systems 5 mM of intracellular ATP completely prevented the develop of UDD, 4 mM was able to partially block UDD and 3 mM ATP induced a modest UDD, as compared to 1 mM ATP (Figure 7). These results suggest that there are differences in the gating of the P2X2R in the 3–5 mM range, suggesting that this receptor can be regulated by changes in cell metabolism.

**Figure 8:**
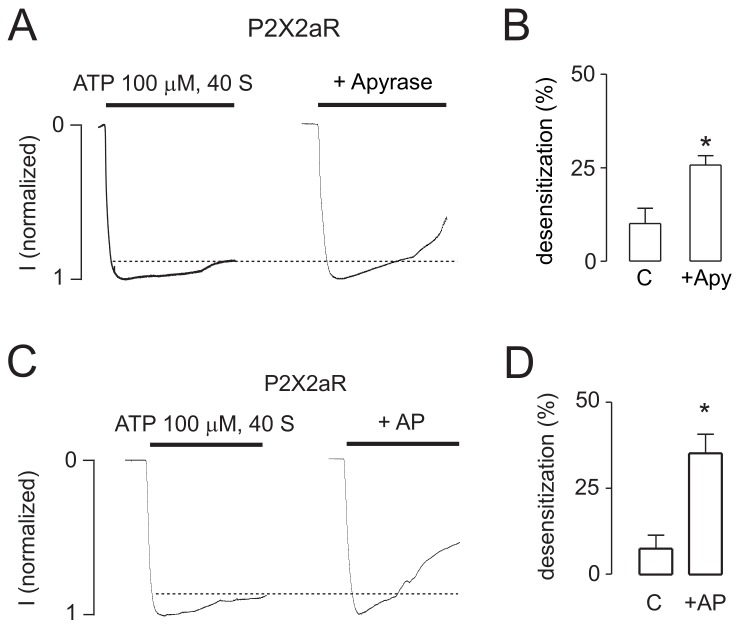
Dependence of the rates of receptor desensitization on the status of intracellular ATP concentration and constitutive phosphorylation of channels. The experiments were performed in oocytes expressing P2X2aR. (**A**,**C**) Representative recordings from oocytes expressing P2X2aR either uninjected (left panels) or injected with 2.5 U/μL apyrase, an ATPase (**A**, right), or 1 U/μL alkaline phosphatase (AP), a hydrolase enzyme responsible for removing phosphate groups from many types of molecules (**C**, right). In both cases, the currents were generated by 100 μM ATP; (**B**,**D**) The mean ± SEM values of the percentage of desensitization after 40 s of ATP application in uninjected cells or those cells injected with apyrase (**B**, *n* = 8) or AP (**D**, *n* = 4); * *p* < 0.01 compared to control columns.

In further experiments, we examined the dependence of the rates of receptor desensitization on the status of the intracellular ATP concentration using TEVC recording in oocytes. To manipulate the intracellular levels of ATP, the oocytes were injected with 2.5 U/μL apyrase, a calcium-activated enzyme that catalyzes the hydrolysis of ATP to yield AMP and inorganic phosphate [35]. Figure 8A shows that injection of this enzyme into the oocytes significantly increased the level of receptor desensitization. Injection of 1 U/μL alkaline phosphatase, a hydrolytic enzyme responsible for removing phosphate groups from many types of molecules [36], also facilitated the desensitization of receptors (Figure 8B). Thus, in intact cells, manipulation of the intracellular ATP levels and the status of kinase/phosphatase activity also influenced the P2X2R desensitization.

### 2.4. The S363 Residue is Critical for the Intracellular ATP Effects on the Channel Gating

The intracellular N- and C-terminal domains of P2X2R contain several residues that could account for constitutive and/or regulated phosphorylation. We utilized the NetPhosk 1.0 Server to predict the residues of P2X2R that could potentially be phosphorylated. Those residues are as follows: Y16, T18, and T372 by MAP kinases; T354, Y362, and S363 by protein kinase C; S377 and S378 by protein kinases C and A; and Y398 by protein kinase A. Because T372, S377, S378, and Y398 are all located on the region of the protein that is absent in the splice variant P2X2bR, and this splice variant also shows UDD and calcium-dependent desensitization [22] (Table 1), we excluded these residues as potential targets for UDD-related phosphorylation and/or dephosphorylation. We then generated mutants of all remaining residues to examine their potential contribution to the development of UDD. We also mutated the D15 and E17 residues because of their potential role in directly coordinating calcium. Finally, we also generated a truncated receptor in which the *C*-terminal Val^439^-Leu^472^ sequence, a sequence that is absent in the fast-desensitizing variant expressed in mouse P2X2eR [22], was deleted. All of the mutants were expressed in HEK293 cells, and the patterns of extracellular ATP-induced currents were examined using the whole-cell recording mode with the potassium-containing intracellular solution. Table 1 summarizes the results obtained with these mutants and with the wild-type P2X2bR.

Substitution of the T18 residue with valine generated a channel that was practically nonfunctional, whereas all other mutants were functional. Several mutants showed significant variations in receptor function compared to the wild type receptor, but UDD was clearly visible in most of the mutants tested, which argued against their roles in intracellular ATP-dependent receptor gating (Table 1). The only exception was the S363 mutant. So next, we studied this residue in more detail.

**Figure 9:**
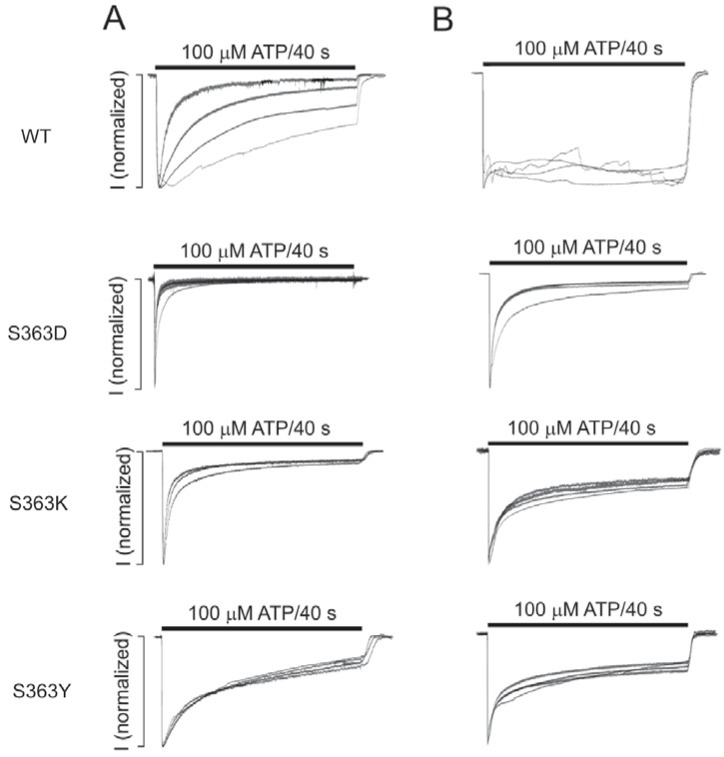
The patterns of extracellular ATP-induced current responses in cells expressing the wild type (WT) and the S363D, S363K, and S363Y mutant receptors of P2X2aR, in the absence (**A**) or in the presence of 5 mM intracellular ATP (**B**). The experiments were performed in HEK393 cells expressing P2X2R at a holding potential of −60 mV. Notice the loss of UDD in all S363-P2X2aR mutants in the absence and presence of intracellular ATP.

We generated five additional S363 mutants: S363C, S363D, S363G, S363K, and S363Y. These mutants in the naïve state showed variable rates of receptor desensitization during the 40 s application of extracellular ATP, but none of them responded with a clearly observable UDD (Table 1, Figure 9). In contrast to the wild-type receptor, addition of intracellular ATP had no influence on UDD. Figure 9 shows the profiles of the current during repetitive 40 s applications of extracellular ATP in the cells expressing S363D, S363K, and S363Y mutants in the absence (left panels) and presence (right panels) of 5 mM intrapipette ATP. The S363D mutant desensitized rapidly in the naïve state, whereas the two other mutants desensitized more slowly, and the rates of receptor desensitization were highly comparable in the presence or absence of intracellular ATP. These results indicate a critical role of the S363 residue in intracellular ATP-dependent regulation of P2X2 and confirm its relevance in UDD.

### 2.5. Role of Mitochondria in P2X2R Desensitization

**Figure 10:**
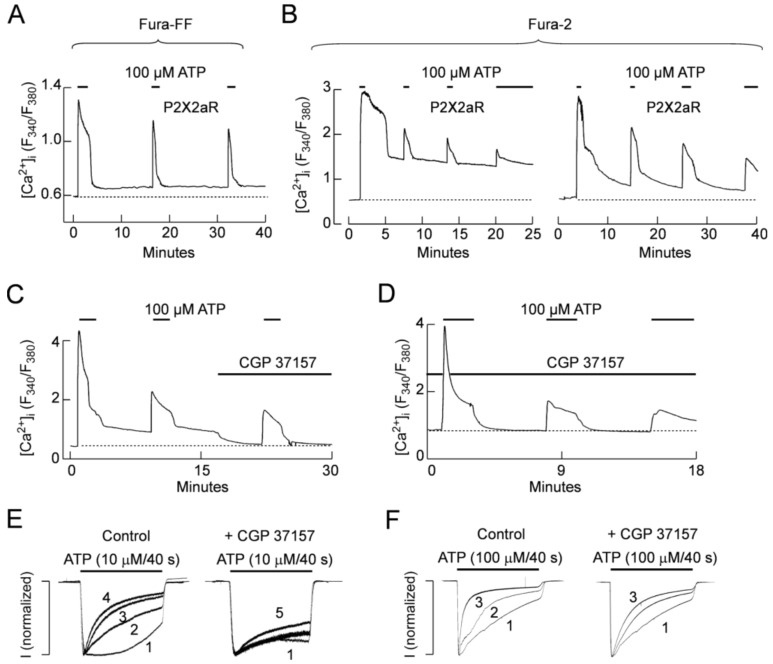
Repetitive ATP application increases basal [Ca^2+^]_i_. (**A**,**B**) Representative [Ca^2+^]_i_ recordings from a P2X2aR-expressing GT1-7 cell in which 3 consecutive ATP application were done (horizontal black bars) with washout period of 15 min between pulses. Recordings were done using Fura-FF (**A**) or Fura-2 (**B**) as Ca^2+^-indicator; (**C**,**D**) Representative [Ca^2+^]_i_ recordings from a P2X2aR-expressing GT1-7 cells in which 4 μM CGP 37157 was applied in the middle (**C**) or during the whole experiment (**D**). Horizontal black bars represent 100 μM ATP applications and dotted lines indicate the basal [Ca^2+^]_i_ before to the first ATP application; (**E**,**F**) Representative recordings from P2X2aR-HEK293 cells in the absence (left) and in the presence (right) of 10 μM CGP 37157; currents were gated with 10 μM (**E**) or 100 μM (**F**) ATP.

It is well established that mitochondria accumulate cytosolic Ca^2+^ at elevated (above 300 nM) concentrations and release it slowly when [Ca^2+^]_i_ drops below this value [37]. Therefore, we further tested the hypothesis that such redistribution of intracellular Ca^2+^ during ATP pulses and interpulse intervals determine basal [Ca^2+^]_i_, can influence in P2X2Rs desensitization. To test this hypothesis, we first performed Ca^2+^ recordings on single intact P2X2aR-expressing cells. In cells loaded with the low-affinity Ca^2+^ indicator Fura-FF, application of 3 ATP pulses caused both UDD and a small but consistent increase in basal [Ca^2+^]_i_ (Figure 10A). To have a better estimation of basal [Ca^2+^]_i,_ we next used cells loaded with the more sensitive Fura-2 indicator; in those experiments we consistently observed an increase in basal [Ca^2+^]_i_ during the washout period (as long as 15 min) in cells expressing the P2X2aR (Figure 10B). Next, we used CGP 37157, a blocker of the mitochondrial Na^+^-Ca^2+^ exchanger (NCX) [38], but also a blocker of plasma membrane NCXs at concentrations >10 μM [38,39]. Application of 4 μM CGP 37157 prevented and reverted the increase in basal [Ca^2+^]_i_ after repetitive ATP applications, indicating that mitochondrial NCXs are responsible for the sustained increase in intracellular Ca^2+^. As shown in Figure 10C, the addition of 4 μM CGP 37157 to P2X2aR-expressing GT1-7 cells completely reverted the increase in basal [Ca^2+^]_i_ observed after an ATP pulse. Moreover, when CGP 37157 was present during the whole experiment no increase in [Ca^2+^]_i_ was observed (Figure 10D). In patch-clamp experiments, pre and co-application of CGP 37157 prevented UDD when P2X2aR-expressing HEK293 cells where stimulated with 10 μM ATP, in the presence of extracellular Ca^2+^ (Figure 10E). However, when 100 μM ATP was used to trigger the currents, CGP 37157 lost its ability to prevent UDD (Figure 10F).

## 3. Discussion

This study summarizes our progress in understanding the regulation of P2X2Rs desensitization by ATP and calcium, and strongly suggests that their gating depends on the metabolic state of the cell. In general, ATP acts extracellularly as an orthosteric agonist for P2XRs, whereas extracellular calcium inhibits or stimulates these channels depending on the subtype of receptors [2]. Several groups observed a strong inhibitory effect of bath calcium on P2X2R, with patterns typical for allosteric mode of regulation [40,41,42]. Calcium also regulates P2XR function by acting as an intracellular messenger. For P2X2R, an interaction with the neuronal calcium sensor VILIP1 was reported [43]. Our recent work with this receptor is focused on UDD [21], which depends on calcium influx and its intracellular action, with domain calcium being sufficient for development of this form of receptor desensitization [22].

The intracellular effect of calcium on receptor desensitization could be allosteric or through activation of some intracellular signaling pathway. For example, in the case of P2X7R, extracellular calcium is an allosteric regulator [44] whereas intracellular calcium acts through CaM [45]. However, CaM was not involved in the intracellular action of calcium on P2X2aR, as indicated by the experiments with the CaM inhibitory peptide and CaM-KII inhibitor KN-93. Additionally, it has been shown that CAM-KII regulates membrane insertion of the P2X3R through direct phosphorylation of the intracellular T388 [46]. It has also been suggested that the protein kinases A and C signaling pathways contribute to the control of P2XR gating [13,47]. Our experiments also suggest that phosphorylation/dephosphorylation have an important role in P2X2aR gating. First, we did not observe UDD in perforated-patch clamp recording, although there is a substantial rise in intracellular calcium concentration under these experimental conditions [48]. UDD was also not present in the TEVC recordings in oocytes, which further suggests that cytosol dilution during whole-cell recordings accounts for the calcium-dependent facilitation of receptor desensitization. Consistent with this, we found that UDD did not occur in cells containing 5 mM ATP in the intracellular solution during whole-cell recordings. The dependence of the receptor desensitization rates on the intracellular ATP concentration was also confirmed using TEVC recording in oocytes. First, injection of apyrase, a calcium-activated enzyme that catalyzes the hydrolysis of ATP to yield AMP and inorganic phosphate [35], significantly increased the level of receptor desensitization. Second, injection of alkaline phosphatase, a hydrolase enzyme responsible for removing phosphate groups from many types of molecules [36], also facilitated receptor desensitization.

The role of intracellular ATP in P2XR gating has not been studied previously, but such mode of regulation is well established for other channels. One of the first channels that are identified as intracellular ATP regulated channel is the CFTR channel involved in pathogenesis of cystic fibrosis [23]. This channel is regulated by phosphorylation of intracellular domains and by allosteric action of ATP [24]. Transient receptor potential (TRP) channels are also strongly influenced by intracellular ATP; TRPV1 is rescued from desensitized state by long action at high concentration ATP [25], whereas TRPC5 is inhibited by both intracellular ATP and its non-hydrolizable analogue AMP-PNP, indicating the allosteric nature of ATP action [49]. The desensitization of TRPV subfamily of channels is also influenced by intracellular calcium [50]. Intracellular ATP also controls gating of inwardly rectifying potassium K_ir_6 channels [26].

Given that ATPγS is a synthetic non-hydrolyzable analog of ATP in which the terminal gamma phosphate cannot be dissociated from the molecule and transferred to substrates, any potential effect of this agonist could only be allosteric. However, this effect was lacking, in our experiments. We also utilized phosphoenolpyruvate, which has the highest thermodynamic propensity to release and transfer its phosphate group to different substrates among the many phosphate-containing species [51], to verify whether a non-enzymatically mediated phosphorylation plays a role in P2X2aR desensitization. UDD did develop in the presence of intrapipette phosphoenolpyruvate, excluding non-enzymatically mediated phosphorylation as a mechanism for the development of UDD. Furthermore, the presence of intrapipette GTP did not interfere with UDD, indicating that ATP selectively regulates this process. The receptor was desensitized in the presence of 5 mM ATP when kinase activity was blocked. Together, these results indicate that ATP does not affect P2X2aR gating directly by binding to an intracellular binding site but, presumably, by the ATP-dependent and kinase-mediated phosphorylation of the channels. Furthermore, a dilution of cytosolic ATP during whole-cell recording, but not in perforated patch-clamp recording, leads to the abolition of metabolic regulation of P2X2R and the development of allosteric effects of calcium.

We can infer from our experiments with PC12 cells that the calcium-induced increase in P2X2Rs desensitization could be of physiological significance. These cells that endogenously express the P2X2R have been extensively used as a model for chromaffin cell function specially in vesicle trafficking and hormone release, processes that are calcium dependent. In undifferentiated PC12 cells P2X2Rs are the main subtype expressed, this has been deducted both from the expression profile [52] and by the functional responses evoked by extracellular ATP [53]. In contrast NGF-differentiated PC12 cells can express different P2XR subtypes [52]. Thus, the rate of P2X2R desensitization will finally have an impact on the total amount of calcium that is entering through this receptor and will finally affect the vesicular release. In this context, the metabolic state of the cell will regulate this release, by affecting P2X2R gating properties. Interestingly, we found that this regulation mechanism can work at intracellular ATP concentrations between 3 and 5 mM, concentrations that could reflect a cell with a low or high metabolism, respectively.

The NetPhosK 1.0 Server (available online: http://www.cbs.dtu.dk/services/NetPhosK/), which predicts protein phosphorylation sites on the basis of eukaryotic kinase specificity [54], suggested the following potential residues of P2X2R that could be phosphorylated: Y16, T18, T354, Y362, S363, S377, S378 and Y398. As previously mentioned, the P2X2bR that lacks the *C*-terminal Val^370^–Gln^438^ sequence can develop UDD, similar to P2X2aR (Table 1) [22]. For that reason, we excluded the residues T372, S377, S378 and Y398 as functionally relevant for the development of UDD. However, this does not rule out the putative role of these P2X2aR-specific residues in other desensitization-related processes; different splice variants of the P2X2R are known to exhibit different desensitization properties [2], and future experiments could help to clarify whether phosphorylation/dephosphorylation could be involved in these differences. Our mutagenesis and electrophysiological studies also investigated residues T18, Y362, and S363 as being potentially relevant for receptor desensitization. The T18V mutant was essentially nonfunctional, precluding further analysis. Moreover, this residue is conserved among all P2XRs subtypes, and because UDD and calcium-dependent desensitization are primarily observed on the P2X2R, it is more plausible that residues specific to this subtype are responsible for UDD. On the other hand, UDD was lost only in S363 mutants, and this effect was independent of intracellular ATP. These data strongly support the hypothesis that the S363 residue is constitutively phosphorylated at high intracellular ATP concentrations, and its dephosphorylation leads to an increase in P2X2R desensitization.

The discovery that the P2X2R gating can be regulated by intracellular calcium and ATP suggests that mitochondria, the source of intracellular ATP and an important regulator of intracellular calcium, could have a key role in this process. For that we run experiments to evaluate the potential role of this organelle in calcium handling during repetitive ATP applications and the potential involvement of such regulation in the desensitization properties of P2X2R using a blocker of mitochondrial calcium transport. CGP 37157 is a preferential blocker of the mitochondrial Na^+^–Ca^2+^ exchanger (NCX) [38,55]. It is well known that mitochondria can regulate [Ca^2+^]_i_ by the action of several transporter proteins [37]. For example, Ca^2+^ uptake is mainly driven by the mitochondrial Ca^2+^ uniporter, whereas Ca^2+^ release to the cytosol is driven by NCX. In this context, the inhibition of mitochondrial NCX by CGP 37157 will cause Ca^2+^ accumulation in this organelle, thereby lowering [Ca^2+^]_i_ and then reverting and/or preventing UDD, as we observed when the currents were gated by low ATP concentrations. Interestingly, when 100 μM ATP was used to gate P2X2aR-currents CGP 37157 was not able to revert/prevent UDD. We infer that the higher calcium influx combined with the leak of intracellular ATP as result of the whole-cell configuration can account for these differential effects. Likewise, in untreated cells, the normal functioning of mitochondrial Ca^2+^ uniporter and NCX will provide a constant and long lasting source of Ca^2+^ to the cytosol, that will lead to increased basal [Ca^2+^]_i_ and therefore UDD will persist under these conditions.

In summary, these data demonstrate the opposite roles of intracellular calcium and ATP in the regulation of P2X2R gating. Intracellular calcium facilitates receptor desensitization in the absence of ATP, whereas in the presence of high ATP, this effect is suppressed, and the receptor operates practically as a non-desensitizing receptor. It is reasonable to conclude that UDD reflects the kinetics of the decay of the intracellular ATP concentration in whole-cell recording. The preservation of the UDD in the presence of various blockers of calcium-dependent enzymes supports the hypothesis that calcium acts allosterically. The loss of UDD in the presence of ATP further suggests that allosteric calcium binding site(s) is (are) not accessible when intracellular ATP is not depleted. In contrast to calcium, intracellular ATP does not act allosterically but is required for receptor phosphorylation. Mutagenesis studies indicated a critical role for the S363 residue as a putative phosphorylation site. Thus, constitutive phosphorylation of P2X2R blocks intracellular calcium-dependent facilitation of receptor desensitization, probably by blocking the allosteric action of calcium. Phosphorylated receptors, a consequence of higher intracellular ATP concentration, are non-desensitizing channels with a high capacity to elevate intracellular calcium for prolonged periods. In contrast, the dephosphorylated receptors with occupied calcium-binding sites, a reflection of a decrease in intracellular ATP concentrations, are rapidly desensitizing channels with limited capacity to maintain elevated intracellular calcium concentrations. Such a dual regulation of P2X2R could be of physiological relevance because the desensitization profiles of the receptors have direct and indirect (through voltage-gated calcium channels) impacts on calcium influx and calcium-dependent cellular processes.

## 4. Material and Methods

### 4.1. P2XR Constructs Used in Experiment

Experiments were performed using the wild type and mutant rat P2X2aRs, and wild-type P2X2bR, which were subcloned into the pIRES2-EGFP vector (Clontech, Mountain View, CA, USA). To generate the mutants, oligonucleotides (synthesized by Integrated DNA Technologies, Rockville, MD, USA) containing specific point mutations were introduced into the rP2X2a/pIRES2-EGFP template using Pfu Ultra DNA polymerase (Agilent Technologies, Santa Clara, CA, USA). A QIAprep Spin Miniprep Kit (QIAGEN, Venlo, The Netherlands) was used to isolate the plasmids for transfection. To identify and verify the presence of the mutations, sequencing was performed by MACROGEN Inc. (Rockville, MD, USA). The P2X2Rs cDNAs were inserted in the pIRES2-EGFP plasmid and expressed in HEK293 cells by polymer-based transfection with the JetPrime transfection reagent (PolyPlus-transfection, Illkirch, France), lipofectamine 2000 (Invitrogen, Carlsbad, CA, USA) or injected into *Xenopus* oocytes.

### 4.2. Receptor Transfection and Cell Culture

The HEK293 cells were routinely maintained in DMEM (Invitrogen, Carlsbad, CA, USA) containing 10% (*v*/*v*) fetal bovine serum (Gibco, Grand Island, NY, USA) and 1% (*v*/*v*) penicillin-streptomycin (Invitrogen, Carlsbad, CA, USA). For the electrophysiological experiments, the cells were grown on 35-mm Nunclon Surface dishes (NUNC, Rochester, NY, USA) at a density of 500,000 cells per 35 mm culture dish. The wild type and mutated receptors were transiently expressed in HEK293 cells by transfection using the JetPrime transfection reagent. Briefly, 2 μL of JetPrime and 2 μL of DNA were mixed in the manufacturer-provided buffer and incubated for 10 min at room temperature. The mixture was then added to the medium covering the cells in a 2 mL Nunclon Surface (NUNC, Rochester, NY, USA) cell culture dish. Alternatively, the transfection was conducted using 2 μg of DNA and 5 μL of Lipofectamine 2000 in 2 mL of serum-free Opti-MEM medium according to the manufacturer’s instructions (Invitrogen, UK or Carlsbad, CA, USA). Experiments were performed 24–48 h after transfection, and the P2X2R-expressing cells were identified by GFP fluorescence.

PC12 cells (derived from pheochromocytoma of rat adrenal medulla, ATCC# CRL-1721) were grown in DMEM containing 10% FBS and penicillin-streptomycin (Invitrogen, Carlsbad, CA, USA). Cells were routinely subcultured and plated on 12-mm poly-l-lysine-coated glass coverslips 1–5 days before electrophysiological recordings.

### 4.3. Patch-Clamp Measurements

Electrophysiological experiments were performed on cells at room temperature using whole-cell patch-clamp recording techniques. The currents were recorded using an Axopatch 200B patch-clamp amplifier (Molecular Devices, Sunnyvale, CA, USA) and were filtered at 2 kHz using a low-pass Bessel filter. Patch electrodes, fabricated from borosilicate glass (type 1B150F-3; World Precision Instruments, Sarasota, FL, USA) using a Flaming Brown horizontal puller (P-87; Sutter Instruments, Novato, CA, USA), were heat polished to a final tip resistance of 2–4 MΩ. All current recordings were captured and stored using the pCLAMP 9 software package in conjunction with the Digidata 1322A analog-to-digital converter (Molecular Devices, Sunnyvale, CA, USA). The bath solution contained (in mM) 142 NaCl, 3 KCl, 1 MgCl_2_, 2 CaCl_2_, 10 glucose, and 10 HEPES (4-(2-hydroxyethyl)-1-piperazineethanesulfonic acid). The Ca^2+^-deficient solution contained (in mM) 145 NaCl, 3 KCl, 10 glucose, and 10 HEPES. To discard the ocurrence of Mg^2+^-dependent unspecific conductances we performed experiments using the Ca^2+^-deficient solution in the absence and in the presence of 2 mM Mg^2+^ (Figure S1). We defined as Ca^2+^-deficient solution because trace amounts of Ca^2+^ (90 μM) were detected in our ultra-purified water by atomic absorption spectrometry using a calcium lamp (AAnalyst 200 Atomic Absorption Spectrophotometer; PerkinElmer, Hongkong, China). In all cases, the pH was adjusted to 7.35, and the osmolarity of these solutions was 295–305 mOsm. Various intracellular solutions were prepared in order to perform these experiments. The sodium-based intracellular solution contained (in mM) 142 NaCl, 10 EGTA, and 10 HEPES. The potassium-based intracellular solution contained (in mM) 140 KCl, 3 MgCl_2_, and 10 HEPES; its pH was adjusted to 7.2 with KOH. Finally, the cesium-based intracellular solution contained (in mM) 154 mM CsCl, 11 mM EGTA, and 10 mM HEPES, adjusted to pH 7.2 with 1.6 M CsOH. ATP was prepared daily in the bath buffer and was applied using the rapid solution changer system RSC-200 (Biologic Science Instruments, Claix, France). Stock solutions of the kinase or phosphatase activators/inhibitors and phosphoinositides were prepared in dimethylsulfoxide, and aliquots were stored at −20 °C. The current responses were recorded from single cells clamped at −60 mV. Perforated-patch recordings were conducted by including 300 μg/mL nystatin in the potassium-containing pipette solution. For intracellular ATP concentration-response experiments we used sodium-based intracellular solutions with 10 mM (HEK293) or 0.05 mM (PC12) EGTA in addition to 0–5 mM ATP and performed UDD protocols.

### 4.4. Oocyte Injection and Current Measurements

Experiments with *Xenopus* oocytes were conducted following the National Institutes of Health guidelines for the care and use of experimental animals and approved (FM N°17, 21 June 2016) by the Bioethics Committee of the Catholic University of the North, Chile. A segment of the ovary was surgically removed from *Xenopus laevis* female frogs under anesthesia (benzocaine, 2 g/L); the oocytes were manually defolliculated and then incubated with collagenase as previously detailed [56]. The nuclei of the oocytes were injected with 3–5 ng of cDNA coding for the various rat receptors using a NanojectII nanoliter injector (Drummond Scientific, Broomall, PA, USA). After 24–48 h incubation in Barth’s solution (in mM: 88 NaCl, 1 KCl, 2.4 NaHCO_3_, 10 HEPES, 0.82 MgSO_4_, 0.33 Ca(NO_3_)_2_, and 0.91 CaCl_2_; pH 7.5; supplemented with 10 IU/L penicillin/10 mg streptomycin and 2 mM pyruvate), additional CaCl_2_ was added to obtain a final 2.5 mM calcium concentration; the Ca^2+^-deficient solution was similar to that used in patch-clamp experiments, but included 1 mM MgCl_2_ to avoid the develop unspecific cationic currents. The oocytes were clamped at −70 mV using the two-electrode voltage clamp (TEVC) configuration with an OC-725C clamper (Warner Instruments, Hamden, CT, USA). The ATP-gated currents were recorded after regular ATP applications repeated every 10 or more minutes depending on ATP concentration. For the experiments with apyrase and alkaline phosphatase, previously P2X2aR-injected oocytes were injected 30 min before the start of recordings with 40 nL of the ectonucleotidase apyrase (2.5 U/μL) or with alkaline phosphatase (1 U/μL). In all cases, we calculated the percentage of desensitization 40 s after the peak amplitude was induced by 100 μM ATP.

### 4.5. Intracellular Calcium Recordings

Transfected GT1-7 cells plated on coverslips were bathed in KR-like medium containing 2.5 μM Fura-FF or Fura-2 AM (Invitrogen) for 1 h at room temperature. After that, the coverslips were washed in KR-like media and they were mounted on the stage of an inverted Observer-D1 microscope (Carl Zeiss, Oberkochen, Germany) attached to an ORCA-ER camera (Hamamatsu Photonics, Hamamatsu City, Japan) and a Lambda DG-4 wavelength switcher (Sutter, Novato, CA, USA). Hardware control and image analysis was performed using Metafluor software (Molecular Devices, Downingtown, PA, USA). Experiments were done under a 40× oil-immersion objective during exposure to alternating 340- and 380-nm excitation beams, and the intensity of light emission at 520 nm (F340 and F380) was followed simultaneously in several single cells.

### 4.6. Data Analysis

Each experiment was repeated in at least four separate cells. Curve fitting and statistical analyses, including nonparametric Mann–Whitney tests, were performed using GraphPad software (San Diego, CA, USA) and Sigmastat (San Jose, CA, USA). Statistical significance was determined using a *p* < 0.01. The desensitization constant (τ) was calculated for each recording by curve fitting of the desensitization phase with a predefined monoexponential function (f(*t*) = B exp(−*t*/*τ*)) using the Clampfit 10.0 software (Molecular Devices, Sunnyvale, CA, USA). Data are reported as mean ± SEM unless stated otherwise.

## Figures and Tables

**Table 1:** Rates of desensitization and maximal currents after repetitive stimulation of the P2X2aR, P2X2bR and mutant P2X2aRs by 100 μM ATP for 40 s followed by 4-min washout periods. The data are presented as the mean ± standard error values, with n of 3–17 per receptor. The P2X2bR is a splice variant lacking the Val^370^–Gln^438^ sequence in the C terminal domain. ΔCR, Truncated receptor with the C-terminal sequence Val^444^–Leu^472^ deleted; n.d. not defined; τ_1_–τ_4_, rates of receptor desensitization and I_1_–I_4_, the peak amplitude of current responses during the four ATP applications. All results are expressed as the mean ± SEM. Nonparametric Mann Whitney-test (*p* < 0.01) was utilized to compare the wild type and mutant receptor amplitude responses and desensitization times. Asterisk (*) denotes significant differences.

Receptor	τ_1_ (s)	τ_2_ (s)	τ_3_ (s)	τ_4_ (s)	I_1_ (nA)	I_2_ (nA)	I_3_ (nA)	I_4_ (nA)
P2X2a	14.6 ± 1.7	7.4 ± 1.8	6.0 ± 1.6	4.3 ± 0.1	2.6 ± 0.5	2.1 ± 0.3	1.4 ± 0.3	0.7 ± 0.1
D15N	14.1 ± 3.2	17.5 ± 1.4 *	6.8 ± 2.5	2.8 ± 0.5	1.4 ± 0.3 *	1.2 ± 0.1 *	0.8 ± 0.1 *	0.56 ± 0.04
Y16D	13.2 ± 2.6	3.6 ± 1.04 *	1.7 ± 0.1 *	1.2 ± 0.4 *	2.1 ± 0.3	0.4 ± 0.1 *	0.2 ± 0.1 *	0.2 ± 0.1 *
E17A	15.2 ± 2.4	5.3 ± 3.0	1.9 ± 0.1 *	n.d.	9.1 ± 0.6	5.7 ± 1.4 *	2.9 ± 0.2 *	n.d.
T18V	0.7 ± 0.1 *	n.d.	n.d.	n.d.	0.2 ± 0.1 *	n.d.	n.d.	n.d.
T354A	12.03 ± 4.6	10.5 ± 1.3	9.7 ± 2.2 *	5.9 ± 1.9	2.2 ± 0.4	0.9 ± 0.3 *	0.8 ± 0.2 *	0.4 ± 0.1 *
Y362A	4.6 ± 1.2	3.9 ± 0.8 *	2.6 ± 0.5 *	0.9 ± 0.4 *	2.1 ± 0.3	0.9 ± 0.1 *	0.6 ± 0.1 *	0.1 ± 0.03 *
S363A	8.4 ± 2.6 *	5.3 ± 1.1	5.1 ± 1.2	4.5 ± 0.9	3.8 ± 0.8	2.8 ± 0.3	2.2 ± 0.6 *	2.4 ± 0.4 *
S363C	3.8 ± 0.5 *	3.1 ± 0.6 *	3.9 ± 0.6	4.8 ± 0.8	1.4 ± 0.3 *	0.4 ± 0.01 *	0.2 ± 0.02 *	0.1 ± 0.01 *
S363K	8.1 ± 2.2 *	7.2 ± 1.8	7.0 ± 1.6	6.9 ± 2.2	1.9 ± 0.6	1.0 ± 0.3 *	0.8 ± 0.3	0.6 ± 0.2
S363D	5.2 ± 1.1 *	4.2 ± 0.9 *	3.4 ± 0.8 *	2.3 ± 0.5	1.9 ± 0.2	1.0 ± 0.2 *	0.9 ± 0.2	0.6 ± 0.2
S363G	7.6 ± 0.9 *	9.6 ± 1.7	9.5 ± 2.9 *	8.2 ± 1.3 *	1.2 ± 0.3 *	1.0 ± 0.2 *	0.7 ± 0.1 *	0.5 ± 0.1
S363Y	8.2 ± 2.8 *	8.7 ± 2.8	6.9 ± 1.7	5.3 ± 1.4	0.7 ± 0.2 *	0.5 ± 0.2 *	0.3 ± 0.1 *	0.2 ± 0.1 *
P2X2b	6.8 ± 1.1	3.5 ± 1.3	2.6 ± 0.5	n.d.	4.8 ± 0.6	4.1 ± 0.5	3.3 ± 0.8	n.d.
ΔCR	22.3 ± 6.9 *	14.5 ± 5.4 *	8.3 ± 0.5	1.5 ± 0.4 *	1.9 ± 0.4	1.5 ± 0.4	0.5 ± 0.1	0.17 ± 0.04

## Supplementary Materials

Supplementary materials can be found at http://www.mdpi.com/1422-0067/19/4/1161/s1.

## Abbreviations

CaM	Calmodulin
P2XRs	P2X receptor channels
TEVC	two-electrode voltage clamp
TRP	Transient receptor potential
