# Supplementary material for: Opposing Roles of Calcium and Intracellular ATP on Gating of the Purinergic P2X2 Receptor Channel
Source: Int J Mol Sci. 2018 Apr 11;19(4):1161. doi: 10.3390/ijms19041161 (PMC5979340; doi:10.3390/ijms19041161)
Supplement: Supplementary file 1 [file ijms-19-01161-s001.pdf]

Supplementary Materials: Opposing Roles of Calcium and Intracellular ATP on Gating of the Purinergic P2X2 Receptor Channel

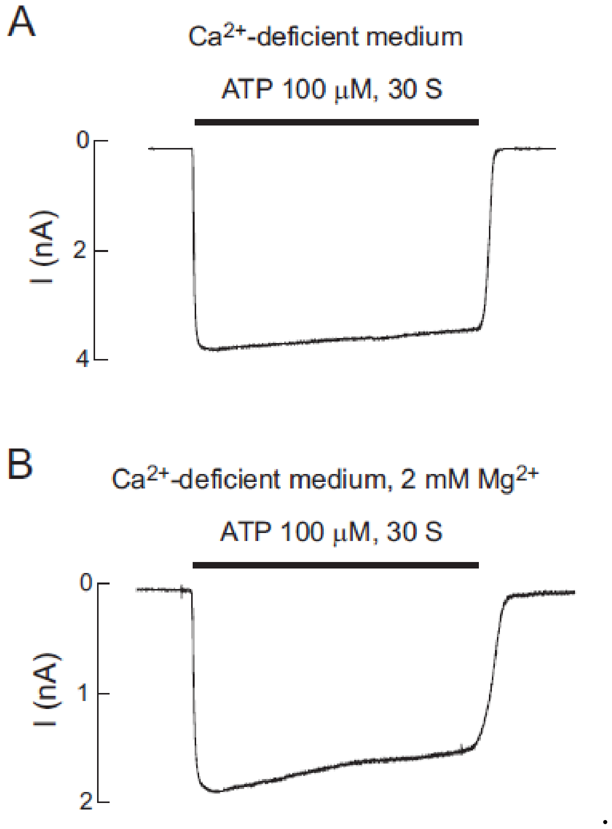

Figure S1. A. Recording from a P2X2aR-expressing HEK293 cell in bathed in a Ca^{2+}-deficient medium with no added of Mg^{2+}. **B**. Recording from another P2X2aR-expressing HEK293 cell in similar conditions to the one shown in **A**, but with the presence of 2 mM Mg^{2+} in the extracellular solution.
